# Establishing essential quality criteria for the validation of circular RNAs as biomarkers

**DOI:** 10.1016/j.bdq.2019.100085

**Published:** 2019-05-02

**Authors:** Christina Pfafenrot, Christian Preußer

**Affiliations:** Institute of Biochemistry, Justus Liebig University of Giessen, D-35392 Giessen, Germany

**Keywords:** Non-coding RNA, Circular RNA, Biomarker

## Abstract

Non-coding RNAs were established in the last decade as a new valuable biomarker class for human diseases. Specifically, circular RNAs (circRNAs) were only recently discovered as a new large group of non-coding RNAs that, due to their circular configuration, are metabolically more stable compared to their linear counterparts and therefore highly suitable for biomarker use. Based on high-throughput sequencing, the catalogs of endogenous circRNAs with disease relevance and correlation continue to grow steadily. As a consequence, circRNAs emerged as novel and attractive biomarkers, indicated by numerous recent publications. Here we would like to stress the need of essential quality criteria for validation and characterization of circular RNAs. In addition to high-throughput sequencing, classical biochemical methods are essential and should be applied for the characterization of this special class of RNAs, in particular to convincingly confirm their circularity.

The rapid evolution of next generation sequencing technologies resulted in the nearly exponential development of putative RNA biomarkers in recent years. In particular, different classes of non-coding RNAs (ncRNA) appear promising for a wide range of clinical applications. This is also reflected in the so-called “Non-coding RNA revolution” [1] and the current focus in non-coding RNA research on investigating the underlying global RNA networks within cells, tissues, or organisms. As a relatively new class of ncRNA biomarkers, circular RNAs (circRNAs) have come more into focus within the last decade (Fig. 1). Although single examples of these particular RNA species had been known for more than forty years [2], circRNAs were established as a large RNA class only a few years ago, based on high-throughput sequencing and bioinformatics [3]. CircRNAs derived from pre-mRNAs (in some cases also from pre-ncRNAs) are characterized by a covalently closed loop structure, which is generated by a special mode of alternative splicing of pre-mRNAs, also called “backsplicing”: A 5′ splice site is joined to an upstream instead of a downstream 3′ splice site (Fig. 2). Some studies also showed that constitutive splice signals as well as the canonical splicing machinery are involved in circularization [4,5]. The average size of a circRNAs can range from under 100 nts to over 4 kb in which the commonly observed circular RNAs consist of 2–3 exons with internal introns removed [6]. Furthermore, circRNA biogenesis can be promoted by *cis*-elements and *trans*-factors by bringing the corresponding splice sites in close proximity, resulting in circularization [7]. In addition to this major, exon-type circRNAs, there are also circular intronic RNAs (ciRNAs) and exon-intron circular RNAs (EIciRNAs) [6]. Although a defined function of circRNAs is still under debate, some studies revealed a microRNA sponge function of naturally occurring circRNAs like ciRS-7 and SRY [8,9]. In addition, several other, hypothetical roles have been discussed, such as templates for translation into peptides or proteins, protein sponging, allostery, scaffold functions in RNA-protein complex assembly, or antisense activity. CircRNAs are cell type-specifically expressed and, due to their structure, very stable compared to their linear counterparts [9]. Because of these biological properties and the correlation with various human diseases, including cardiovascular diseases [10,11], disorders of the nervous system [12,13], diabetic retinopathy [14] and cancer [15], it becomes clear that circRNAs may play an important role as specific biomarkers. Since circRNAs are also found in extracellular vesicles circulating in various body fluids such as blood and saliva this should greatly extend the potential of circRNAs as prognostic and diagnostic biomarkers, especially for liquid biopsies. Specifically, RNA-Seq reads spanning the backsplice junction allow the genome-wide bioinformatic identification of circRNAs. However, we observe a certain amount of different and facile approaches to unequivocally prove circularity of this important ncRNA class. This comprises all stages of circRNA identification, ranging from RNA-Seq approaches, bioinformatics, to biochemical validation. Here we would like to stress that stringent quality criteria have to be defined, which distinguish real circular configuration from possible artifacts.

**Fig. 1:**
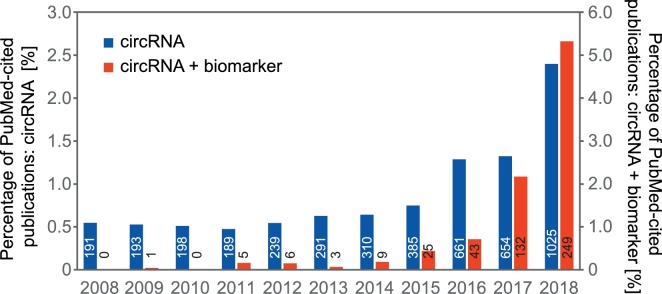
Circular RNA as emerging novel biomarkers. The rapid development of circRNA research and circRNA in combination with biomarkers can be inferred from the percentage of PubMed-cited publications (https://www.ncbi.nlm.nih.gov/pubmed) using *circular RNA* (blue graphs) and *circular RNA* + *biomarker* (red graphs) as keywords in searches, of publications using the general terms *RNA* and *RNA* + *biomarker* as keywords, for publications between 2008 and 2018. The number inside the bars represents the absolute number of publications.

**Fig. 2:**
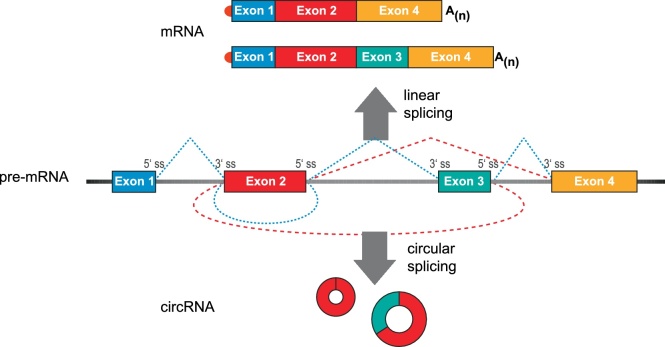
Biogenesis of circular RNAs (circRNAs). Schematic representation of the biogenesis of linear mRNA and circular RNA. During canonical splicing, introns are removed and exons are joined with each other, generating either a single mRNA from the pre-mRNA (constitutive splicing) or several splice variants (alternative splicing). CircRNAs are generated by an alternative splicing mechanism, also referred to as backsplicing, in which a 5′ splice site (5′ ss) is joined to an upstream 3′ splice site (3′ ss) instead of a downstream 3′ ss. Different splice variants can be generated by alternative backsplicing resulting in single- or multi-exonic circular RNAs (circRNAs). Exons are depicted as colored boxes and introns as solid lines. The 5′ cap structure of mRNA is shown as red dots and the 3′ poly(A) tail as A(n). Constitutive (blue) and alternative splicing (red) is indicated by dashed lines.

Already the initial stage of identification of novel circRNAs requires the establishment and standardization of unbiased approaches. Several things have to be considered: The RNA integrity is critical, since neither RT-PCR might detect circRNAs molecules nor other methods such as Northern blotting, since the characteristic circular junction might get lost during partial degradation [16]. Even the choice of library preparation itself has a strong impact on the results. In particular, one of the first fundamental steps during RNA-Seq library preparation is crucial, namely the reverse transcription. Most of the reverse transcriptases used are known to be error prone and can frequently introduce mutations, which have been widely recognized [17]. As a result, aberrant *trans*-splicing products can be generated during reverse transcription, mainly based on a template-switching activity of some enzymes [18,19]. This phenomenon can also occur in the case of linear transcripts, but in particular for circRNAs can easily result in misinterpretations.

In addition, endogenous *trans*-spliced transcripts can lead to false positive events. In particular, duplicated exon sequences within one mRNA (intragenic *trans* splicing) or transcripts derived from different genes on different chromosomes (intergenic *trans* splicing) can be detected and result in false positive events [20]. Another critical step during library preparation is that most of the commercially available RNA-Seq library kits are designed to analyze either the RNA profile of long RNAs, such as mRNA and lncRNAs, or of small ncRNAs, for example miRNA. Again, this general limitation applies also to circRNAs. Furthermore, the library preparation from circRNAs either requires initial fragmentation to generate accessible 5′ and 3′ ends, or relies on random hexamer priming, which may result in inefficiency and/or biased representation in RNA-Seq [21]. This turns out to be especially challenging for the analysis of RNA profiles from extracellular vesicles, which primarily contain short RNAs less than 500 nts, however longer transcripts were reported as well [22,23].

After sequencing, the large number of available circRNA prediction tools can introduce additional variability. Some of them require an established annotation and some knowledge about existing exon-intron structures, while others, such as CIRI [24], rely on *de novo* assembly and thus are able to identify new backsplice junctions. These different options of evaluation tools can result in a dramatic differences between the outputs of algorithms used [21]. Referring thereto, a recent study compared 11 different circRNA detection tools on stimulated and on real datasets and could show that CIRI, CIRCexplorer and KNIFE achieved better balanced performance between their precision and sensitivity compared to other methods [25]. Nevertheless, many of these algorithms show strongly aberrant results and a high degree of false positive results, for example in relation to their resistance to RNase R treatment. These studies show that CircRNA detection tools should be treated with care, and that a combination of tools is required to make reliable predictions [21]. Therefore one should consider the combination of more than one method for the bioinformatic analysis to minimize the number of false positives.

However, from our point of view, most critical is the often neglected stringent biochemical validation of the circRNAs, which should follow the bioinformatic evaluation. Unfortunately, in many recent publications, including many on biomarker detection, this important and crucial issue is restricted to a minimum. As a method of choice, RNA-Seq and the bioinformatic analysis in combination with RT-(q)PCR approaches are employed as the only assays for validation. RT-qPCR approaches have the great advantage – in particular digital PCR- to check genome-wide data sets in a quantitative manner and represents the simplest and fastest method to detect the circularity of circRNAs. By designing divergent primer pairs relative to the circularizing exon/s, PCR products can be generated (usually 100–200 nts), which specifically include the characteristic circular splice site. Beyond the scope of this point of view, this method offers certain limitations, which are particularly relevant in the validation of circRNAs and are mainly based on the problems caused by the transcriptases as already mentioned above. One possibility to circumvent this problem is to combine the RT-PCR analysis with RNase R treatment. RNase R is a 3′ to 5′ exoribonuclease that degrades linear RNA, whereas most circRNAs are resistant due to their circular configuration. Unfortunately, relatively large circRNAs tend to be not absolutely RNase R resistant and in addition, complex secondary structures and chemical modifications in linear RNAs can influence the RNase R activity [16]. Moreover, variations in RNases R activities among different batches and manufacturers, experimental settings, as well as secondary RNA structure, might affect the efficiency of linear RNA removal as well.

To unequivocally demonstrate a circular configuration, Northern blot analysis, which is RNase R independent, should be defined as the gold standard and also as a method of choice (for details on the method, see [26]). Although this classic type of RNA analysis is often neglected because it is somewhat more time-consuming and requires some practical experience, Northern blotting is greatly versatile and thereby ideally suitable for circRNA characterization. For Northern blot detection, probes are designed that either span the backsplice junction or hybridize to the total transcript. By simply choosing a suitable gel-electrophoresis system (agarose and/or polyacrylamide), the two possible configurations (circular or linear) can be clearly distinguished from each other.

Furthermore, Northern blotting can also be combined with RNase R or RNase H ribonuclease digestion. In particular, the RNase H cleavage assay has emerged as an elegant method for validation of circRNAs [4,27]. In brief, antisense oligonucleotides are designed directed against a specific circRNA. RNase H recognizes DNA-RNA hybrids and subsequent cuts the RNA within the duplex. The characteristic shift of mobility during denaturing polyacrylamide gel electrophoresis from an aberrantly slow migration of the circRNA to the expected linear behavior, as well as the cleavage patterns of RNase H digestion, can then be analyzed by Northern blotting. Furthermore, new approaches should be considered for a biochemical validation of the circular configuration. Here, among other things, probe-based systems (*in situ* hybridization) could play a crucial role. Moreover, current developments in next-generation sequencing such as the nanopore technology (Oxford Nanopore Technologies) should likely provide new options in the future. MinION’s for example could provide emerging direct RNA sequencing capabilities.

In sum, all the problems mentioned here can only be circumvented by combining several biochemical validation methods and by maintaining stringent quality standards. Especially in the field of biomarker development, complete and convincing circRNA characterization should be a top priority. The commonly mentioned low reproducibility and the variable analytical standards are often an obstacle during the development of potential new biomarkers. CircRNAs highlighted here, indeed harbor great biomarker potential, but there is a strong need for standardization and for setting up minimal requirements for validating this class of ncRNA. How could this be achieved? The growing circRNA community should define what is essential to establish a new circRNA species as such. In recent years this has been successfully implemented for other issues, such as the MIQE (minimum information for publication of quantitative real-time PCR experiments) [28] and MISEV (minimal information for studies of EVs) [29], where the respective communities and their experts have provided recommendations on experimental methods and minimal information in reporting.
